# The Vitamin D Binding Protein and Inflammatory Injury: A Mediator or Sentinel of Tissue Damage?

**DOI:** 10.3389/fendo.2019.00470

**Published:** 2019-07-10

**Authors:** Richard R. Kew

**Affiliations:** Department of Pathology, Stony Brook Cancer Center, Renaissance School of Medicine, Stony Brook University, Stony Brook, NY, United States

**Keywords:** inflammation, neutrophil accumulation, chemotactic factor, vitamin D binding protein (DBP), tissue injury

## Abstract

Neutrophils are the most abundant type of white blood cell in most mammals including humans. The primary role of these cells is host defense against microbes and clearance of tissue debris in order to facilitate wound healing and tissue regeneration. The recruitment of neutrophils from blood into tissues is a key step in this process and is mediated by numerous different chemoattractants. The neutrophil migratory response is essential for host defense and survival, but excessive tissue accumulation of neutrophils is observed in many inflammatory disorders and strongly correlates with disease pathology. The vitamin D binding protein (DBP) is a circulating multifunctional plasma protein that can significantly enhance the chemotactic activity of neutrophil chemoattractants both *in vitro* and *in vivo*. Recent *in vivo* studies using DBP deficient mice showed that DBP plays a larger and more central role during inflammation since it induces selective recruitment of neutrophils, and this cofactor function is not restricted to C5a, as prior *in vitro* studies indicated, but can enhance chemotaxis to many chemoattractants. DBP also is an extracellular scavenger for actin released from damaged/dead cells and formation of DBP-actin complexes is an immediate host response to tissue injury. Recent *in vitro* evidence indicates that DBP bound to G-actin, and not free DBP, functions as an indirect but essential cofactor for neutrophil migration. DBP-actin complexes always will be formed regardless of what initiated an inflammation, since release of actin from damaged cells is a common feature in all types of injury and DBP is abundant and ubiquitous in all extracellular fluids. Indeed, these complexes have been detected in blood and tissue fluids from both humans and experimental animals following various forms of injury. The published data strongly supports the premise that DBP-actin complexes are the functional neutrophil chemotactic cofactor that enhances neutrophil chemotaxis *in vitro* and augments neutrophilic inflammation *in vivo*. This review will assess the fundamental role of DBP in neutrophilic inflammation and injury.

## Historical Context and Brief Background

The vitamin D binding protein (DBP, also abbreviated VDBP) was initially named the group-specific component of serum (abbreviated as Gc-globulin). Jan Hirschfeld is credited with the first publication in 1959 describing the migration pattern of an unknown serum protein using agarose gel electrophoresis and rabbit anti-human serum (i.e., immunoelectrophoresis) (1). This report described qualitative differences in the electrophoretic migration of α_2_-globulin proteins in serum obtained from 10 normal healthy blood donors (1). These immunoreactive proteins would display a consistent migration pattern (either fast, intermediate, or slow) that was specific to an individual donor, and thus was named the group-specific component of serum (1). This paper, together with two subsequent papers by Hirschfeld published the following year (2, 3), initiated this six decade long investigation into the structure and function of DBP. A major milestone for DBP research occurred in 1975 when Diager et. al. reported that the serum group-specific component binds vitamin D, and consequently, the authors proposed to name the protein the vitamin D binding α-globulin (4), later shortened to the vitamin D binding protein. A few years later several reports described what initially was thought to be a larger cellular form of DBP (5–7) but subsequently was shown unequivocally to be plasma DBP binding to G-actin monomers in a 1:1 molar complex (8). Thus, by 1980, DBP was known to have two distinct binding functions: vitamin D and G-actin. Structural studies would later confirm the vitamin D sterol and G-actin binding pockets in DBP (9, 10). Beginning in the mid-1980's and continuing into the early 1990's, numerous reports identified DBP and plasma gelsolin as part of the actin scavenging system in blood. These proteins work in tandem to clear extracellular actin released from dead or damaged cells for removal from the circulation, largely in the liver (11). Furthermore, several studies demonstrated that serious injury (burns, traumatic injury, acetaminophen-induced hepatotoxicity) generate large amounts of extracellular actin that consumes DBP and reduces its plasma concentration (12–15). Low levels of plasma DBP directly correlate with poor overall survival in these studies (12–15). Although the actin binding properties of DBP have been clearly established and shown to be physiologically relevant, this role largely has been overshadowed by the vitamin D sterol binding function, particularly over the past 15 years with increasing awareness of the essential role of vitamin D in health and disease. Besides, the vitamin D binding protein is not the best choice of name to promote its actin binding function! There are many facets of this multifunctional plasma protein, several of which are discussed in other reviews of this series. The topic herein will focus primarily on the actin binding capacity of DBP, how this function plays a role in chemotaxis enhancement of neutrophils and may act as a possible mediator of inflammatory tissue injury.

## DBP-Actin Complexes and Tissue Injury

Tissue injury often causes cell damage and death leading to the release of intracellular contents into extracellular fluids. Many intracellular molecules (HMGB1, mitochondrial DNA, ATP, etc.) have different roles in the extracellular environment and function as “alarmins” (also known as danger-associated molecular patterns or DAMPs) to signal the immune system to the presence of tissue injury (16). Although numerous alarmins have been described and their role in tissue injury well-characterized, it is quite surprising that very little attention has been paid to actin, the major cytoskeletal protein that is the most abundant intracellular protein in any organism. Actin essentially exists in two states inside a cell: monomeric globular actin (G-actin), or G-actin polymerized into filaments (F-actin) (17). These two forms are in a constant state of polymerization and depolymerization that is highly regulated by several intracellular actin binding proteins, and this dynamic process is very prevalent in motile cells such as leukocytes (18). During tissue injury large quantities of actin can be released into extracellular fluids where the protein escapes normal intracellular regulatory mechanisms and the protein spontaneously will form F-actin filaments. In animal models, extracellular F-actin filaments have been shown to alter the coagulation and fibrinolytic systems leading to occlusion and damage of the microcirculation (particularly in the lung) in a manner similar to how fibrin damages the microvasculature in disseminated intravascular coagulation (15, 19). Accordingly, an effective extracellular scavenging system has evolved to dispose of actin released from dead or damaged cells (15). The system is composed of two plasma proteins that have complementary functions: gelsolin, which severs F-actin filaments into G-actin monomers, and DBP that binds G-actin in a high affinity (K_*d*_ of 10^−9^ M) 1:1 molar complex for transport and eventual clearance primarily in the liver. This process consumes DBP and a decreased plasma concentration of actin-free DBP has been shown to be an effective but indirect marker of tissue injury in cases of severe trauma in both humans and animal models (15). Although there has been interest in the potential therapeutic role of gelsolin as an extracellular actin scavenger to treat several conditions, less attention has been focused on its partner DBP, despite the fact that over the past 30 years numerous studies (>400 listed on PubMed) have reported that DBP is associated with various acute and chronic diseases in several different organs [reviewed in (20, 21)]. There is no apparent common connection in these various studies, but a very broad view of all these reports would suggest that DBP has a fundamental role during tissue injury, perhaps due to its actin scavenging function.

The vitamin D binding protein is an abundant plasma protein that is part of the albumin gene family and shares considerable amino acid homology and structural similarity with these proteins (11). Several groups have published the crystal structure of DBP that revealed an α-helix triple domain arrangement (characteristic of the albumin family) that forms a broad U-shaped or saddle-shaped molecule (9, 22, 23). The N-terminal (domain I) and the C-terminal domains (III) form the front and back of the saddle and domain II the seat (10, 22, 23). This shape is designed to perfectly fit a molecule of G-actin, and this has been confirmed by crystal structure analysis of DBP bound to G-actin (10). Vitamin D sterol binding pocket in domain I is distinct from the actin binding domain, and a molecule of DBP can bind both ligands simultaneously without apparent alteration in binding affinity or protein function (24). Although DBP mRNA expression has been reported in many different tissues, plasma DBP is synthesized by hepatocytes in the liver and circulates in blood with a range of 5–9 μM (300–500 μg/ml) (11). It has a relatively rapid turnover in plasma with a half-life of about 2 days (compared to 22 days for albumin) (25). In contrast to albumin whose plasma levels decrease during inflammation, DBP levels in blood are stable or rise slightly during the acute phase response of inflammation (26). Moreover, DBP is ubiquitous *in vivo* and significant quantities (0.1–1 μM range) have been detected in all extracellular fluid compartments (cerebrospinal, bronchoalveolar, synovial, etc.) (25, 26). Although DBP is the primary transport molecule for vitamin D sterols in the blood and extracellular fluids, its concentration in plasma is much higher than vitamin D. Normally only 1–2% of the total circulating DBP pool has vitamin D bound and this percentage never rises above 5% (27). This is in contrast with other transport proteins in blood where about 50% of the total protein pool is bound with ligand (25). This fact prompted many to speculate that DBP must have other essential functions, perhaps related to its ability to scavenge G-actin (28). Indeed, significant tissue injury can result in a large percentage of circulating DBP complexed with actin (12–15). Actin-induced depletion of plasma DBP to levels below 3.5 μM (200 μg/ml) have been shown to be an effective but indirect marker of tissue injury that correlates with poor prognosis in cases of sepsis, multiple trauma and acetaminophen-induced liver failure (12–15). Clinical outcome and decreased plasma levels of DBP have a statistical correlation similar to the other outcome metrics such as the APACHE II score (sepsis), Kings College criteria (liver failure) and the TRISS-like method (multiple trauma) (13–15).

The vitamin D binding protein and actin are abundant and ubiquitous proteins of the intra- and extracellular compartments, their expression is stable during inflammation and cell injury causes immediate complex formation. Thus, global transcriptome analysis of mRNAs increased or decreased during inflammatory injury most likely would not identify actin or DBP (29). For these reasons we believe that the potential role of DBP-actin complexes during inflammation has been overlooked. Varying levels of DBP-actin complexes are continually formed *in vivo* as a result of minor tissue trauma, menstrual cycles, infections, surgery, etc. Hence, low levels of circulating DBP-actin would be a routine physiological process and should be transient and inconsequential. On the other hand, prolonged generation and/or high concentrations of DBP-actin in extracellular fluids potentially could act as a danger signal (alarmin) of ongoing and significant tissue injury. Although previous research has focused on actin-free DBP in plasma, the role of DBP-actin complexes in tissue injury has not been determined, most likely because it has been assumed that these complexes are inactive by-products of cell damage that are rapidly cleared from the circulation. Interestingly, a previous study examining fulminant hepatic necrosis (FHN) in humans showed that 72% of plasma DBP was complexed with actin in patients who died of the disease whereas only 22% of total DBP was bound to actin in FHN survivors (12). However, while this data was very compelling, the authors of this study focused on actin-free DBP, not the level of DBP-actin complexes. Although DBP-actin complexes generally have been viewed as benign by-products of cell injury, recent studies from our lab (both *in vitro* and *in vivo*) have shown that these complexes may serve as an alarmin and possess a cytokine-like function. Thus, the accumulated evidence has shown that DBP, via its actin binding function, likely plays a role in both the mediation and resolution of tissue injury.

## Cell-Associated DBP

The vitamin D binding protein interacts with many different cell types to achieve its diverse functions (delivery of vitamin D sterols, binding and/or clearance of G-actin, chemotactic cofactor function, macrophage activating-factor), and it appears that the protein must first bind to its target cell surface in order to mediate these effects. Numerous studies have reported DBP on the surface of most cell types including B-lymphocytes (30–32), T-lymphocytes (33), testicular cells (34), placental cytotrophoblasts (35, 36), pancreatic acinar cells (37), monocytes (38), neutrophils (39), and renal proximal tubule cells (40). It is clear that cell-associated DBP is not a novel cellular form but rather circulating DBP bound to the cell surface (41). Although binding to the plasma membrane is the first step in the interaction of DBP with cells, it is unlikely that a cell surface binding site for DBP would be a specific high affinity (*K*_d_ ≤ 10^−8^ M) receptor. DBP is abundant and ubiquitous in all fluid compartments and circulating blood leukocytes are bathed in 5–9 μM DBP (11). Therefore, a relatively low affinity binding site with a *K*_d_ significantly above the plasma concentration of DBP would seem most logical, otherwise leukocytes in blood could act as a “sink” for DBP and diminish the circulating pool. Several studies have shown that DBP binds with low affinity to megalin, also known as LDL receptor related protein 2 (LRP2) (42–44). Megalin is a large scavenger receptor that is primarily expressed on the surface of epithelial cells in the kidney (proximal tubules), endocrine glands, and reproductive organs (45). Megalin on renal proximal tubules binds and captures DBP (both free and bound to vitamin D) in glomerular filtrate for re-uptake into the circulation (45). DBP also binds specifically, but with low avidity, to chondroitin sulfate glycosaminoglycans (CS-GSGs), and more specifically to the CS-GAG group on CD44, which is widely expressed on most leukocytes (46, 47). However, several papers have reported that DBP bound to the surface of cells cannot be removed by numerous high salt and/or detergent washes, suggesting a relatively tight binding site (30, 33, 38).

Our lab has extensively investigated the binding of DBP to human neutrophils and myeloid cell lines (U937, HL-60) since cell binding is the critical first step in the process of DBP functioning as a chemotactic cofactor. DBP does not follow the kinetics of saturable binding to a single high-affinity receptor, but instead displays a multiphasic, time-dependent interaction with neutrophils over the course of 60 min at 37°C: weak binding (5–20 min), strong binding (20–50 min), shedding into the extracellular fluid (>45 min) (46). There is very little (if any) binding of radiolabeled DBP to cells incubated in an ice bath (1°C), but at 37°C three distinct phases of DBP binding are observed, indicating that a dynamic process is required to express this binding site. In addition, if neutrophils are first stimulated with chemotactic factors or calcium ionophores at 37°C prior to incubation with labeled DBP, the delay in tight binding phase is essentially eliminated. The initial weak binding of DBP to neutrophils probably is mediated by low avidity molecules such as CD44 or other chondroitin sulfate proteoglycans upregulated from internal stores in neutrophil cytoplasmic granules (46, 48). These weak binding molecules may serve to capture DBP from extracellular fluids and loosely tether it to the cell surface.

The biochemical characterization of the tight binding site for DBP on the plasma membrane of neutrophils, and mechanisms of the subsequent protease-mediated shedding, were very extensive (46, 48–50) and showed that: (a) DBP bound to the plasma membrane can only be dissociated using harsh denaturing conditions; (b) solubilization of cells revealed that the bound DBP partitions to the detergent insoluble fraction, which contains the actin cytoskeleton; (c) confocal immunofluorescence microscopy revealed that DBP and actin co-localize on the surface of neutrophils; (d) immunoprecipitation of DBP bound to (or shed from) cells, followed by mass spectrometry analysis showed that the major binding partners were actin, CD44 and annexin A2. Annexin A2 is known as a molecular facilitator since it binds multiple diverse molecules (including CD44 and actin) to assemble plasma membrane complexes in a phospholipid and calcium-dependent manner (51). Interestingly, two reports published more than a dozen years before our studies on neutrophil cell surface DBP binding site, described that DBP was tightly bound to actin on the surface of human B lymphocytes (30) and monocytes (38).

The third phase, DBP shedding from the cell surface, was shown to be due to the action of the enzyme elastase on the DBP binding site, since DBP is not modified (cleaved) during this process (49). Elastase is a physiologically important protease with broad substrate specificity, its expression is restricted mainly to neutrophils and abundant quantities of this protease are stored in the azurophil (primary) granules in the cytoplasm of both immature and mature neutrophils (52). Inhibition of elastase will inhibit shedding and cause DBP to accumulate on the plasma membrane, and also will inhibit the chemotactic cofactor function of DBP (49). Previous reports have described proteolytically active elastase on the cell surface of neutrophils *in vitro* (53, 54). Interestingly, both DBP binding, and shedding of the binding site, are constitutive processes and occur in the absence of an inflammatory or chemotactic stimulus, however these stimuli accelerate the process (46, 49). Moreover, as will be discussed below, DBP binding to cell surface actin on neutrophils and subsequent elastase-mediated shedding from the plasma membrane, may be a key step in understanding its function as a chemotactic cofactor.

In addition to DBP uptake from fluids, we also have previously reported that human neutrophils contain an intracellular store of DBP in specific (secondary) granules (39), and this finding was verified in a follow-up study investigating the DBP binding site in neutrophils (46). Neutrophil specific granules contain a diverse array of molecules including several binding proteins such as haptoglobin and vitamin B_12_ binding protein (55). It is not clear why these cells have DBP stored in intracellular granules formed during myelocyte stage of neutrophil development in the bone marrow (56). Perhaps cells utilize this store of DBP during chemotaxis and phagocytosis when there is dynamic rearrangement of cell structures and release of granule contents (56). The amount of DBP was calculated to be 3 ng/10^6^ neutrophils, a rather small quantity but considering that an inflammatory exudate may contain billions of neutrophils, the amount of DBP that neutrophils potentially could release in an inflammatory lesion may not be insignificant.

## Neutrophils, Inflammatory Injury, and the Chemotactic Cofactor Function of DBP

Neutrophils are the primary “rapid response” cells of the innate immune system that are essential for host defense (57, 58). Individuals with a marked reduction in circulating neutrophils (neutropenia), either due to a rare congenital abnormality or more commonly a consequence of cancer chemotherapy, are highly susceptible to severe and often life-threatening bacterial and fungal infections. The importance of maintaining adequate numbers of circulating neutrophils is highlighted by the fact that ~60% of the total bone marrow output of all blood cells is dedicated just to produce neutrophils (59). It is estimated that the average healthy person has a steady-state production of 100 billion neutrophils per day under homeostatic conditions, whereas cell production increases significantly and rapidly during an infection, a process known as emergency granulopoiesis (60, 61). However, it has been well-known for more than a century that excessive tissue accumulation of neutrophils is observed in many inflammatory disorders (neutrophilic inflammation). Cytotoxic products from activated neutrophils mediate significant tissue injury and are linked to the pathogenesis of numerous acute and chronic diseases (57–59). The destructive potential wrought by excessive numbers of responding neutrophils is highlighted by the fact that this cell type can liquify and obliterate tissue, i.e., a cavity resulting from an abscess (62). There has been remarkable progress over the past 5–8 years in understanding various aspects of neutrophil biology, from their birth in the bone marrow to their death at sites of inflammation and everything in between. Perhaps most interesting is that neutrophils possess considerable plasticity and are far more adaptable to their environment than previously thought. Furthermore, neutrophils can display several disparate functions that contradict their long-time moniker as “masters of tissue destruction.” Many excellent reviews on various aspects of neutrophil biology have been published recently (63–66).

Migration of neutrophils from the bloodstream into various tissues is a critical stage during inflammation triggered by both infectious and non-infectious stimuli (63). Although much is known about the chemoattractants and their receptors that initiate and direct the neutrophil migratory response, little is known about factors in physiological fluids that regulate tissue recruitment of these cells. More than 35 years ago several investigators had demonstrated that normal human serum possesses a heat stable chemotactic enhancing activity for complement activation peptide C5a (67–69). A 60 kDa protein (called co-chemotaxin) was partially purified from human serum and shown to be capable of enhancing the neutrophil chemotactic activity of C5a and its stable degradation product C5a des Arg (70). Previously, we were the first of several groups to identify that DBP was the serum co-chemotaxin for C5a (71, 72), and several other groups subsequently confirmed our initial observations that DBP can augment the chemotactic activity of C5a/C5a des Arg (73–79). These studies all reported that DBP does not possess chemotactic activity but requires chemoattractants to exert its cofactor activity. Other studies also reported that DBP is not able to augment other C5a-mediated functions in neutrophils such as oxidant generation and degranulation (release of cytoplasmic granule contents) (71, 79). But most curious was the observation that DBP could not enhance the chemotactic activity of other major neutrophil chemoattractants: formylated peptides, CXCL8 (IL-8), leukotriene B_4_ or platelet activating factor (71, 72), leading to the general consensus that the chemotactic enhancing properties of DBP appeared to be restricted to C5a/C5a des Arg. All of these reports used an *in vitro* chemotaxis assay, employing either blind-well or Boyden chambers, to measure an increase in neutrophil migration when DBP was added. Although this assay is very sensitive and quantitative, it has limitations that resulted in the mistaken conclusion that DBP was specific for complement peptide C5a. C5a is a very potent neutrophil chemotactic factor and on a molar basis is 10–50 times more potent than other major chemoattractants when tested *in vitro* (71, 80). Therefore, the chemotactic enhancing activity of DBP during the short incubation (30 min) filter-based assays was particularly noticeable when C5a was used as the stimulus. However, utilizing a different *in vitro* assay (under agarose), that requires a longer incubation period (4 h), revealed that DBP could enhance neutrophil movement to other chemoattractants as well (81). Moreover, *in vivo* studies using the DBP null mice (discussed below) have shown that the chemotactic cofactor activity of DBP is not specific for C5a as previously thought but can augment the chemotactic activity of perhaps many leukocyte chemoattractants. These recent *in vitro* and *in vivo* studies described below have helped to better define how DBP functions to enhance neutrophil chemotaxis that may contribute to neutrophilic inflammation.

## Lessons From the DBP Null Mouse

The initial reports describing the chemotactic cofactor function of DBP were received enthusiastically at that time in the inflammation research community (71, 72). However, that high level of interest faded with the subsequent failure to describe a clear mechanism of chemotaxis enhancement, and to provide confirmation that DBP enhances neutrophil chemotaxis *in vivo*. Previous attempts to define a mechanism using *in vitro* approaches were not successful. For example, our lab performed numerous chemical cross-linking and co-immunoprecipitation experiments that demonstrated clearly DBP does not physically-associate with the C5a receptor (50). In addition, we reported that and DBP does not bind to C5a, and cell binding of C5a and DBP are independent events (82). Other groups have shown that DBP does not alter the number of neutrophil C5a receptors or the receptor *K*_d_ for C5a, thereby discounting another obvious explanation for its co-chemotactic effect (79, 83). The question of physiological relevance and whether DBP enhances neutrophil recruitment to tissues *in vivo* needed to wait more than 10 years until a DBP null mouse was generated.

The vitamin D binding protein has been very well-studied in multiple different populations worldwide for over 50 years, but no natural homozygous deficiency of DBP had been reported in humans, or to the best of our knowledge, any mammal. There was no clear reason why a DBP-deficiency was never identified but it was widely speculated that a DBP deletion may be embryonic lethal. Nevertheless, Nancy Cooke's lab at the University of Pennsylvania produced the first mice that were homozygous null for DBP (84). These mice were healthy, of similar size and appearance as wild-type mice, and the DBP null males and females were fertile and produced normal sized litters (84). This phenotype was somewhat surprising at the time given the fact that a natural DBP deficiency had not been observed. Analysis of blood chemistry values revealed that DBP null mice had essentially the same levels of serum calcium, phosphorus, PTH, and alkaline phosphatase as DBP sufficient wild type mice when fed a standard vitamin D replete mouse chow diet (84). However, the serum of DBP null mice had >95% reduction in both 25(OH) and 1,25 (OH)_2_ vitamin D (84). Moreover, when placed on a vitamin D deficient diet, the DBP null mice quickly developed secondary hyperparathyroidism and bone mineralization defects such as osteoid thickening that were not seen in DBP sufficient wild-type mice (84). The initial DBP null strain was backcrossed onto a C57BL/6J background for 10 generations and was used in further studies which showed that the lack of circulating DBP does not alter the tissue distribution, uptake, activation or biological potency of vitamin D (85). Thus, DBP does not alter bioavailable vitamin D but appears to function as a circulating reservoir of 25(OH) vitamin D.

Since no case of homozygous deficiency in humans has been reported, a long-standing unresolved question has been: can DBP null mice function as a murine model for DBP deficiency in humans? Surprisingly, during the preparation of this review, the first report of a DBP deficient human was published (86). The individual is a 58 year-old female who presented with long-standing and progressive ankylosing spondylitis and severe vitamin D deficiency that did not respond to vitamin D supplementation. Laboratory results showed a deletion of the DBP (*GC*) gene and corresponding absence of circulating DBP. Although this individual had a profound deficiency in both 25(OH) and 1,25 (OH)_2_ vitamin D, her blood calcium levels were normal (86). The bone abnormalities and blood chemistry values of this DBP deficient woman were similar to those observed in DBP null mice fed a vitamin D-deficient diet (84, 86). *In vitro* studies using dermal fibroblasts from this DBP null patient showed that DBP does not alter cellular uptake of bioactive vitamin D and expression of the responsive gene *CYP24A1*, a very similar result also was previously reported using cells from DBP null mice (85). However, no information was provided about this patient's response to infections or tissue injury. This report provides some degree of validation that DBP null mice can act as a model for DBP deficiency in humans.

## DBP Null Mice and Inflammatory Injury Models

Our *in vivo* studies of inflammatory injury utilized the DBP null mouse strain developed by Nancy Cooke's lab (84, 87). This DBP null strain was re-derived the by *in vitro* fertilization using DBP^−/−^ sperm and a wild-type C57BL/6J female (DBP+/+) to produce DBP^+/−^ hemizygotes which were cross-bred to generate the DBP null (^−^/^−^) and wild type (+/+) mouse colonies (81). These DBP^−/−^ and DBP^+/+^ mice have been used in three injury models: acute lung injury, multiphasic (acute, chronic, fibrotic) lung injury, and acute muscle injury (81, 88). Each model clearly showed that DBP null mice always have less inflammation, and most noticeably, significantly fewer neutrophils at the site of injury. The markedly reduced neutrophilic inflammation observed with DBP null mice is very consistent among the injury models and reproducible between experiments, but the underlying cellular and molecular mechanisms that are responsible for generating this phenotype is only partially understood. A proposed tentative model is described in a separate section below.

The first study to examine the *in vivo* role of DBP in inflammatory injury used a model of acute complement-dependent alveolitis, induced by either immune complexes or purified mouse C5a (81). In both alveolitis models, DBP null mice had significantly reduced (~50%) neutrophil recruitment to the lungs compared to their wild-type DBP^+/+^ counterparts, and lung histology showed significantly less inflammation in the null mice (81). Another important observation is that addition of exogenous DBP to the lungs of DBP null mice completely rescued their neutrophil recruitment defect. The same study also showed that bronchoalveolar lavage (BAL) fluid from wild-type mice had extensive DBP-actin complexes (~75% of total DBP) 4 h after induction of alveolitis. Although as predicted there were no DBP-actin complexes in DBP null mice, there was detectable actin in the BAL fluid from these animals, indicating actin release from damaged cells (81). These results indicate that DBP null mice have impaired neutrophil recruitment due to lack of DBP and not a cellular defect since the total number, receptor expression and chemotaxis of circulating DBP null neutrophils are essentially identical to cells from their wild-type DBP^+/+^ counterparts.

A second study investigated if a systemic DBP deficiency could attenuate multiphasic lung injury and tissue remodeling induced by bleomycin (81). Wild type and DBP null mice received bleomycin by oropharyngeal aspiration; lung injury was evaluated after 7, 16, or 21 days. DBP null mice all survived to day 21 and did not display overt signs of morbidity whereas all wild-type mice died between day 13 and 16 and showed clear signs of respiratory distress. Bronchoalveolar lavage (BAL) fluid from wild-type mice had extensive DBP-actin complexes (60–75% of total DBP) whereas DBP null mice had no complexes but had evidence of free actin. Analysis of BAL fluid on days 7 and 16 post-treatment showed that both mouse strains had similar numbers of lung macrophages and lymphocytes, but DBP null animals had significantly fewer lung neutrophils. Histological analysis of the lungs on day 16 showed that DBP null mice had a 50% decrease in fibrosis and collagen deposition as compared wild-type animals. This study demonstrated that a systemic deficiency in DBP provides significant protection from bleomycin-induced inflammation and fibrosis in mice.

The third *in vivo* study utilized an acute muscle injury model induced by injection of 50% glycerol into the thigh muscle (88). All animals survived the procedure, but intramuscular glycerol injection showed lysis of skeletal myocytes, and inflammatory cell infiltrates in both strains of mice. The muscle inflammatory cell infiltrate in DBP null mice had remarkably few neutrophils as compared to wild-type mice. The neutrophil chemoattractant CXCL1 was significantly reduced in muscle tissue from DBP null mice. Plasma obtained 48 h after glycerol injection revealed that DBP null mice had significantly lower levels of systemic cytokines IL-6, CCL2, CXCL1, and G-CSF. Multiplex analysis of 36 cytokines indicated that DBP null mice had a less inflammatory and more pro-reparative cytokine profile than their wild-type DBP^+/+^ counterparts (88).

These *in vivo* studies comparing null mice to wild-types showed that DBP may have a central role during inflammation since it induces selective recruitment of neutrophils, and the DBP cofactor function is not restricted to C5a as prior *in vitro* studies indicated, so the physiological implications are much broader. So how does DBP contribute to inflammation by enhancing neutrophil recruitment? The accumulating *in vitro* and *in vivo* evidence appears to suggest that DBP-actin complexes may act as an alarmin and trigger pro-inflammatory functions. DBP is the major extracellular scavenger for actin released from damaged/dead cells and formation of DBP-actin complexes is an immediate host response to tissue injury. All of the *in vivo* injury models discussed above had evidence of DBP-actin complexes only in wild-type mice, and DBP repletion in the acute alveolitis model reversed the neutrophil recruitment defect in the DBP null mice. The currently accepted actin scavenger hypothesis states that injury-induced depletion of plasma DBP will diminish the actin binding capacity in blood and extracellular fluids, leading to formation of actin filaments that obstruct flow and damage small blood vessels (15). Furthermore, if the current actin scavenger hypothesis is correct, it follows that DBP null mice should succumb rapidly to an intravenous bolus of actin which would obstruct the pulmonary vasculature. However, contrary to what would be predicted by the actin-free DBP hypothesis, we observed that DBP null mice do not succumb to a bolus of purified actin injected intravenously (89). In fact, DBP null mice were largely resistant to the lung inflammation and injury observed in wild-type mice, and the null mice appeared utilize plasma gelsolin to clear the actin bolus. Wild-type mice also had a large percentage of their total plasma DBP pool (78%) bound to actin 1.5 h after i.v. injection. Moreover, *in vitro* studies showed that purified DBP-actin complexes added to cultured endothelial cells caused direct cell damage (at 4 h) or death (at 24 h), providing clear evidence that DBP-actin complexes have a direct detrimental effect on cells (89). Thus, results from DBP null mice provide strong evidence to refute the prevailing actin scavenger hypothesis and suggest that the inverse hypothesis may be valid, i.e., an increase in DBP-actin complexes, and not a reduction in actin-free DBP, correlates with inflammation and injury.

## A Possible Role for Vitamin D in Inflammatory Injury?

Vitamin D is known to regulate numerous genes that are involved in the immune and inflammatory response. *In vitro* and *in vivo* studies have shown that active vitamin D produces a tolerogenic, anti-inflammatory, and reparative phenotype as evidenced by immune cell activation status and cytokine profiles (90, 91). DBP null mice have almost no detectable serum vitamin D but actually are vitamin D sufficient when fed a vitamin D replete chow diet (84, 87). Although plasma DBP does not alter the tissue availability of vitamin D, the effect on immune cells in the blood is not known. The lack of plasma DBP in mice may permit greater delivery of bioactive vitamin D to immune cells during their transit in the blood, potentially altering their transcriptomes to dampen inflammation and limit tissue damage. Superimposed on this possible scenario of transcriptional regulation by vitamin D is a lack of DBP-actin complexes during tissue injury. Perhaps both mechanisms together cause DBP null mice to have less neutrophilic inflammation and resultant tissue damage following injury. However, these possibilities remain to be investigated. Finally, it is interesting to note that we previously reported that bioactive vitamin D (1,25 dihydroxy-vitamin D_3_) bound to DBP at physiologically relevant concentrations of 10 and 100 pM, completely abolished the DBP chemotactic cofactor function of human neutrophils *in vitro*, but had no effect on chemotaxis to optimal concentrations of four different chemoattractants (80). In contrast, 25-hydroxy-vitamin D_3_ bound to DBP had no effect on the chemotactic cofactor function (80), thus providing evidence of a direct inhibitory effect of bioactive vitamin D on the DBP chemotactic cofactor function for neutrophils.

## Proposed Model of DBP-Actin Complexes and Inflammatory Injury

Recent evidence indicates that DBP bound to G-actin, and not free DBP, functions as an indirect but essential cofactor for neutrophil migration, thus, providing a possible mechanism to explain how DBP functions to enhance neutrophil migration *in vitro* and recruitment to sites of inflammation *in vivo* (81, 88, 89). However, it is not clear how DBP-actin complexes enhance neutrophil recruitment and inflammation. It is interesting to speculate that DBP-actin complexes may augment a chemotactic signal and cause release of other proinflammatory molecules stored within neutrophils. Perhaps the most attractive candidate in this scenario is calprotectin, a 24 kDa heterodimer composed of S100A8 and S100A9 that can be rapidly released from neutrophils (92). Abundant quantities of S100A8 and S100A9 are stored in the cytosol of neutrophils, and upon cell activation these molecules can be released into the extracellular space as active heterodimers or heterotetramers (92). S100A8/A9 has multiple proinflammatory functions and has been shown to mediate both neutrophil bone marrow development and facilitate chemotaxis of mature circulating cells, most likely by binding to toll-like receptor 4 (TLR4) on the plasma membrane (92). The proposed mechanism involving DBP-actin complexes has clear physiological relevance since DBP is abundant and ubiquitous in all fluid compartments, and release of G-actin from damaged/dead cells is a consistent feature in all types of inflammatory injury. Moreover, we have previously reported that DBP binds to actin on the neutrophil plasma membrane followed by elastase-mediated shedding of these complexes, perhaps as microvesicles, into the extracellular fluids (46, 48, 49). We propose that DBP-actin complexes can bind (or re-bind following shedding) to a neutrophil surface receptor that triggers S100A8/A9 release. In turn, S100A8/A9 binds in an autocrine or paracrine manner to neutrophil TLR4 inducing a signal that synergizes with the chemoattractant receptor signal to enhance migration. Furthermore, it is well-known that TLR4 ligation and signaling synergizes with signals from chemoattractant receptors to enhance leukocyte chemotaxis both *in vitro* and *in vivo* (93, 94).

This provisional model may explain how a deficiency of DBP results in significantly decreased neutrophilic inflammation. However, there are several “unknowns” with this model that need to be investigated. First, the putative receptor for binding DBP-actin complexes has not been identified, but other studies have shown that the receptor Clec9A binds F-actin and is involved in sensing damaged cells (95–97). It is not known if the Clec9A receptor also binds G-actin complexed with DBP or if even if this receptor is expressed on neutrophils. Second, it is not known if the putative DBP-actin receptor signals release of S100A8/A9 when it is ligated with complexes. Third, a question remains do all DBP-actin complexes function the same or are there modifications that differentiate between inflammatory and benign complexes. Finally, do other cell types besides neutrophils and endothelial cells also respond to DBP-actin complexes, particularly hepatocytes and Kupffer cells in the liver (the primary site of DBP-actin clearance).

## Future Directions

Many questions about the functions of DBP remain to be answered. However, new investigative tools and experimental approaches will be needed to decipher these functions. For example, new mouse models with tissue specific inducible expression of DBP, or a mouse model constructed with selective deletions in functional regions within DBP (vitamin D, actin, cell binding regions) by utilizing CRISPER-Cas9 gene editing technology. Another essential tool currently needed is an antibody that only recognizes a neoepitope on DBP-actin complexes, and not DBP or G-actin monomers. This antibody then could be used to develop an ELISA that specifically detects only DBP-actin complexes in biological fluids. In closing, research into the biological functions of DBP has progressed far since Hirschfeld's initial description 60 years ago, but perhaps in the near future new experimental approaches using advanced technologies (single cell transcriptomics, mass cytometry, advanced microscopy, and *in vivo* imaging, etc.) and bioinformatic analysis may reveal what DBP actually has been doing all along.

## Author Contributions

The author confirms being the sole contributor of this work and has approved it for publication.

### Conflict of Interest Statement

The author declares that the research was conducted in the absence of any commercial or financial relationships that could be construed as a potential conflict of interest.
